# Advances and Prospects in Understanding Vertebrate Cardiac Conduction System, Pacemaker Cell, and Cardiac Muscle Development: Toward Novel Biological Therapies

**DOI:** 10.3390/muscles2040026

**Published:** 2023-10-12

**Authors:** Ridwan Opeyemi Bello, Shannon Frew, Yusra Siddiqui, Rashid Minhas

**Affiliations:** 1 School of Human Sciences, College of Science and Engineering, University of Derby, Derby DE22 1GB, UK; 2 Faculty of Health and Life Sciences, University of Exeter, Exeter EX4 4QJ, UK

**Keywords:** cardiac conduction system, pacemaker, gene regulatory network

## Abstract

The heart is composed of muscle cells called cardiomyocytes, including a specialized population named pacemaker cells that form the cardiac conduction system (CCS), which is responsible for generating the action potential dictating heart contractions. Failure of the CCS system leads to cardiac arrhythmias, which require complicated therapies and often the surgical implantation of electrical pacemakers. However, recent research has focused on the development of novel therapies using biological pacemakers that aim to substitute electrical devices. While most signaling pathways and transcription factors involved in the development of the pacemaker cells are known, the upstream regulatory networks need to be predicted through computer-based databases, mathematical modeling, as well as the functional testing of the regulatory elements in vivo, indicating the need for further research. Here, we summarize the current knowledge about the vertebrate myocardial CCS system and the development of the pacemaker cells, as well as emphasize the areas of future research to clarify the regulation of muscle pacemaker cells and the ease of development of biological therapies.

## 1. Contracting Cardiac Muscle

**Figure 1:**
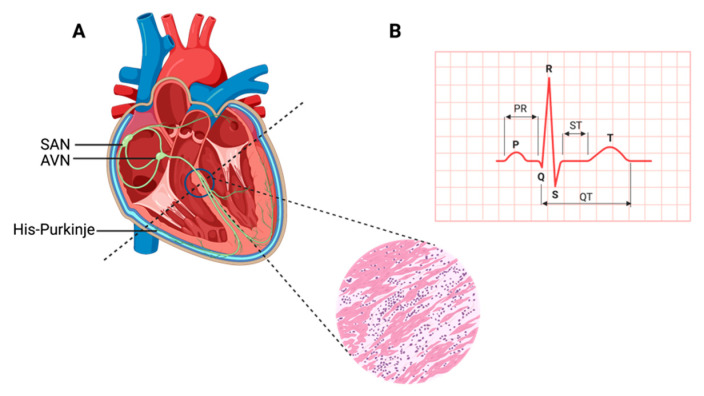
Cardiac contraction and the histology of cardiac muscle. (**A**) Schematic representation of the components of the cardiac conduction system (CCS) in a human heart. In the inset picture, a cross-section of the human heart muscle is shown with binucleated cardiomyocytes. The various components of the CCS (in green) are labeled: the sinoatrial node (SAN), found at the junction of the superior caval vein and right atrium, generates the impulse that then travels to the atrioventricular node (AVN). Propagation occurs through the left and right bundle branches of His-Purkinje, leading to ventricular contraction. (**B**) An electrocardiogram representing the recording of the electrical activity of the heart. The upper chambers of the heart (atria) begin to beat when the first wave of the ECG, labeled P, appears. The lower chambers of the heart (ventricles) are represented by the QRS complex as an electrical current flow. The electrical current spreads back over the ventricles in the opposite direction during the recovery phase, which is represented by the T wave.

The heart, a muscular organ, orchestrates blood circulation throughout the body by generating and coordinating electrical impulses. These impulses are controlled by the cardiac conduction system (CCS), which consists of myogenic components that regulate the contractions of the atria and ventricles [1,2,3,4]. In higher vertebrates, the CCS is divided into specific regions: the slow-conducting structures, such as the sinoatrial node (SAN); the primary site for pacemaker cardiomyocytes (CMs), often referred to as the “natural pacemakers” of the heart, which are located between the superior vena cava and the right atrium; the secondary pacemaker, the atrioventricular node (AVN), which is situated within the atrioventricular septum; and the fast-conducting ventricular conducting system (VCS), which includes the atrioventricular bundle (AVB, also known as the His-Bundle), the right and left bundle branches (BBs), and the Purkinje fiber network (PFN) (which is responsible for ventricular coordination) [3,5,6]. Pacemaker cells within the CCS are a specialized population with a unique and vital role. They have the remarkable capability to spontaneously generate regular electrical impulses, effectively setting the pace for the entire heart. These rhythmic depolarization and repolarization cycles in pacemaker cells are responsible for initiating each heartbeat and maintaining its regularity [4]. These electrical signals rapidly travel through the atrial cardiomyocytes, thus initiating atrial contraction [7]. Subsequently, they navigate through slower-conducting tissues within the AVN, introducing a deliberate delay before transmitting to the His-Purkinje fibers, thereby effectively coordinating ventricular contraction [4,8] (Figure 1).

Cardiac muscle development is a complex and tightly regulated process involving the differentiation and specialization of various cell types, including CMs and pacemaker cells. Notably, diverse vertebrate species exhibit a remarkable spectrum of cardiac development timelines. In a developing human embryo, the initial indications of heart muscle contractions typically emerge around embryonic day 22, which marks the third week of gestation. This coincides with the evolution of the first heart field into the heart tube [9]. In comparison, mice exhibit an earlier onset of heart muscle contractions, typically occurring around embryonic days E8 to E9 [10,11]. However, zebrafish exhibit a unique timeline, possessing distinct heart muscle anatomy from humans, mice, or chickens. Contraction in zebrafish begins as early as 22 h after fertilization. In chickens, contraction initiates at approximately HH10 to HH11 [12,13,14], and each species reflects the intricacies of its cardiac development.

Studies from mammalian, avian, and fish model systems have shown that each CCS component consists of a specialized group of CMs with distinctive morphological and electrophysiological properties, as well as transcriptional profiles [15]. This specialized development of the CCS and pacemaker cells plays a pivotal role in establishing a fully functional heart. However, disruptions or defects in this developmental process can lead to cardiac arrhythmias, including conditions like Brugada syndrome, long QT syndrome, and sudden cardiac death [16,17]. These arrhythmias are characterized by irregular or slow heartbeats, thus ultimately compromising the heart’s ability to efficiently pump blood. Importantly, some of these arrhythmias can further complicate the treatment of congenital cardiac conditions, which often require therapeutics like ion channel blockers, or surgical interventions such as ablation or electronic pacemaker implantation [4]. However, the limitations associated with conventional pacemakers have highlighted the pressing need for alternative pacemaker solutions, leading to the emergence of biological pacemakers as a promising avenue for improving the management of cardiac arrhythmias. 

## 2. Emergence of Biological Pacemakers

In recent years, a remarkable shift in cardiac research has focused on the groundbreaking concept of biological pacemakers, whereby the aim is to harness the intrinsic capacity of the heart to generate electrical impulses. These innovative therapies entail the conversion or manipulation of existing cardiac cells into pacemaker-like counterparts, thus eliminating the reliance on external electronic devices. This research trend has gained significant momentum owing to its potential to address the limitations associated with conventional pacemakers while offering a transformative approach to cardiac rhythm management [18].

Conventional pacemakers have undeniably been instrumental in treating cardiac arrhythmias and ensuring proper heart function. However, their reliance on battery power, limited lifespan, and potential complications from invasive implantation procedures have underscored the need for alternative approaches [18,19]. The emergence of biological pacemakers seeks to overcome these challenges by exploiting the natural regenerative potential of the heart.

The regenerative capacity of the adult mammalian heart, though constrained by the restricted turnover of cardiomyocytes [20], has ignited promising avenues of research. Studies involving neonatal mouse and, potentially, human hearts have unveiled a primitive regenerative capability. Neonatal cardiomyocytes have demonstrated the remarkable ability to re-enter the cell cycle, thereby contributing to the regeneration of damaged myocardia [21,22,23,24]. This regenerative insight, combined with the genetic manipulation of specific signaling pathways and innovative cell therapies, has emerged as a promising approach for facilitating myocardial recovery post-injury.

While substantial strides have been made in comprehending cardiac differentiation from diverse cell sources, including embryonic stem cells (ESCs), induced pluripotent stem cells (hiPSCs), and adult cardiac stem cells (CSCs) [25,26,27,28,29], a notable gap in knowledge persists in the role of the cardiac conduction system. Despite extensive investigation into the major cardiac cell types like cardiomyocytes, fibroblasts, endothelial cells, and cardiac stem cells during myocardial regeneration, the intricate orchestration of the cardiac conduction system has remained relatively understudied [22,30,31]. This deficiency in understanding can be attributed to a series of significant limitations within the field, which each contribute to the relative lack of exploration in this domain:

(1) Limited Conduction Cell Numbers: The scarcity of conduction cells within the heart presents a considerable challenge. (2) Intra- and Intercomponent Heterogeneity: The diverse array of cell types and components that constitute the cardiac conduction system introduces challenges in elucidating the distinct roles and interactions of each element (3) Challenges in Isolation: Isolating pure populations of conduction cells for in-depth study remains a formidable task. (4) Complex 3D Anatomy of the CCS: The cardiac conduction system’s 3D anatomy, which involves intricate networks of cells and structures, as well as further complicates efforts to decipher its functionality [32].

At the core of the development of biological pacemakers lies the intricate process of reprogramming the existing cardiac cells into pacemaker-like entities [33]. This multifaceted endeavor necessitates the precise orchestration of gene expression and signaling pathways to induce a pacemaker-like phenotype within non-pacemaker cardiomyocytes [33]. A comprehensive understanding of the underlying molecular mechanisms governing pacemaker cell development is pivotal, as it unveils potential targets ripe for genetic manipulation.

Despite a century of studying heart development, the formation of pacemaker cells remains a realm of limited exploration due to the intricate genetic mechanisms underpinning their development. To propel biological pacemaker research forward, it is imperative to translate existing knowledge of signaling pathways, transcription factors, and gene regulatory networks into tangible reprogramming efforts.

## 3. Key Transcription Factors Involved in CCS Development

Exploring CCS development and homeostasis is reliant on transcriptional and regulatory networks that are embryonic-stage-dependent, dose-dependent, and tissue-dependent [34,35,36]. A cascade of transcription factors, SHOX2, BMP4, NKX2-5, ID2, ISL1, GATA4, HAND1, IRX3, and various T-box transcriptions factors are instrumental to the divergence in myocyte development. Below are sub-sections that show the evidence as to why these transcription factors are important.

### 3.1. Short Stature Homeobox 2 (SHOX2) and Bone Morphogenic Protein 4 (BMP4)

SHOX2 and BMP4 are recognized as pivotal factors contributing to the formation of the SAN, a critical element in heart rhythm regulation [4,37]. Dysfunction of the SAN can precipitate various cardiac arrhythmias, including bradycardic arrhythmias [38]. The SHOX2 transcription factor holds essential significance in both SAN development and differentiation. Its wide expression throughout the body, including the heart muscle, underscores its multifaceted role [38]. Conversely, BMPs, a subgroup of signaling molecules within the Transforming Growth Factor β superfamily, play a vital role in pacemaker development. BMP4, in particular, assumes a central position in embryonic heart development by promoting fibroblast reprogramming into cardiomyocytes with pacemaker activity [39]. Notably, SHOX2 exerts influence over BMP4, where their expressions overlap [40]. Among the remarkable functions of BMP4 are that it takes a lead role in driving the differentiation of cardiac pacemaker cells [41]. Illumination from epistatic genetic experiments conducted in Xenopus has unveiled a direct interaction between SHOX2 and the BMP4 promoter. The closely coordinated expression patterns of BMP4 and SHOX2 are especially conspicuous in the SAN during embryonic development [40].

### 3.2. T-Box Transcription Factor 5 (TBX5), NK2 Homeobox 5 (NKX2-5), and Inhibitor of DNA Binding 2 (ID2)

TBX5, NKX2-5, and ID2 play indispensable roles in the development of the atrioventricular bundle and bundle branches [4,42]. The transcription factor TBX5, while having diverse functions across the body, holds a pivotal role in cardiac development [42]. Mutations in TBX5 have been associated with cardiac defects in the septa and CCS. During early embryonic cardiac development, TBX5 functions as a transcriptional activator for genes involved in cardiomyocyte maturation [42]. In later cardiac development stages, TBX5 shifts its focus to the structure of the CCS and the maintenance of cardiomyocyte maturation [42]. NKX2-5, a cardiac homeobox transcription factor with an expression spanning the cardiac system, plays a crucial role in regulating cardiac development and function [43]. Mutations in NKX2-5 result in cardiac defects and atrioventricular conduction irregularities. Throughout cardiac development, NKX2-5 is instrumental in regulating the function of working and in conducting myocytes within the atria, often in coordination with the Notch signaling pathway [43]. ID2, another cardiac transcription factor, is initially detected in areas like the neural crest, in inflow and outflow tracts, and in neurons around the aorta and pulmonary artery [44,45]. In later developmental stages, ID2 expression becomes apparent in the atrioventricular bundle around E12.5 and subsequently in the bundle branches by, approximately, E16.5 [42,45].

### 3.3. T-Box Transcription Factor 3 (TBX3)

TBX3, a vital player within the CCS, is integral for repressing atrial differentiation and maintaining proper cardiac function [46]. Various studies have linked noncoding variants near TBX3 expression to alterations in PR interval and QRS duration, underscoring its impact on atrioventricular conduction [47,48,49,50,51,52]. TBX3 is primarily found in the SAN, which is a part of the heart’s electrical system. It plays a major role in regulating the genes active in the SAN while actively suppressing genes associated with atrial function, ensuring that the SAN retains its pacemaker function and does not become atrial tissue [4]. When TBX3 is introduced where it is not typically found, it prompts the development of functional pacemaker cells within the atria [53,54]. In essence, TBX3 transforms regular cardiac cells into pacemaker-like cells within its domain of influence.

In the nearby developing atrial heart tissue, Nkx2-5 has an opposing role. Nkx2-5 represses the expression of TBX3 and another gene called Hcn4. This is consistent with observations in embryos lacking Nkx2-5, which show abnormal expressions of TBX3 and Hcn4 in the heart tube [55]. Conversely, introducing extra Nkx2-5 into heart muscle cells, including those in the SAN, prevents the proper formation of the SAN [56]. This indicates that Nkx2-5 acts to confine the influence of TBX3 and Hcn4 to specific areas of the heart.

Interestingly, the absence of Nkx2-5 in the SAN, while present in other heart muscle cells, provides a valuable tool for identifying SAN cells in laboratory-grown human ESCs. When scientists coax human ESCs into becoming heart cells, they produce both cells similar to those found in the heart’s chambers (NKX2-5+) and pacemaker-like cells that lack NKX2-5 expression (NKX2-5−) [57]. This research enhances our understanding of cardiac development and the roles of these critical transcription factors.

### 3.4. T-box Transcription Factor 18 (TBX18)

TBX18 plays a pivotal role in heart muscle development, particularly in shaping the structure and formation of the SAN [41,58]. Its expression is essential for early SAN specification, and it generates pacemaker activity during the initial phases of embryonic heart muscle formation [46,59,60]. Surprisingly, when Tbx18 is deficient in mice (which leads to underdeveloped sinus venosus and SAN structures), these mice do not display significant bradycardia (slow heart rate). Intriguingly, even in the presence of this deficiency, the SAN gene program remains intact in their underdeveloped SANs. This suggests that while Tbx18 may not directly control the SAN gene program, it plays an essential role in ensuring the proper formation and deployment of progenitor cells [46]. However, when Tbx18 is artificially introduced into ventricular myocytes via a viral method, it has a distinct impact. Specifically, it reduces the expression of connexin 43 (Cx43), a protein responsible for gap junction intercellular communication between cells, which regulates cell death, proliferation, and differentiation (while not affecting Cx40 and Cx45), in these ventricular myocytes [61]. In the ventricles of pigs and guinea pigs, the introduction of Tbx18 leads to a phenomenon called ‘reprogramming,’ where ventricular myocytes start to exhibit pacemaker-like properties and generate ectopic pacemaker activity. Alongside this reprogramming, there is a suppression of Cx43 and natriuretic peptide A (Nppa), as well as an increase in Hcn4 expression [62,63]. It is speculated that the differences observed between the loss and gain of Tbx18 function experiments can be attributed to the fact that Tbx18, which primarily acts as a repressor T-box factor [64], mimics the function of Tbx3 when overexpressed [4].

### 3.5. ISLET-1 (ISL1)

ISL1, a transcription factor, fulfills diverse roles across multiple organs during embryonic development, and, within cardiac development, it serves as a marker for second heart field progenitors [65]. Its expression is detectable as early as E7 in mouse heart development, and its pattern shifts as development progresses, with the expression being observed in the SAN from postnatal stages through to adulthood [66]. In zebrafish, Isl1 is a marker for pacemaker cells located at the junction of the sinus venosus and atrium, where it is necessary for normal pacemaker function and development [67]. In mice, Isl1 is indispensable for the proliferation and proper functioning of SAN cells, and its specific deletion within the SAN results in embryonic lethality [37]. Notably, the absence of Isl1 in mice leads to the downregulation of the key regulators involved in SAN development, such as TBX3, SHOX2, and BMP4, as well as ion channels that are crucial for SAN function, including HCN4, HCN1, and Cacna1g [37,68]. Conversely, when Isl1 is overexpressed in the cardiomyocytes derived from ESCs, it upregulates the genes associated with the SAN while downregulating genes linked to chamber myocardia [69]. Remarkably, Isl1 is a target of SHOX2 within the SAN, and it can rescue the bradycardia phenotype that results from SHOX2 deficiency [70]. This emphasizes the pivotal role of ISL1 in the development and regulation of pacemaker cells within the heart’s SAN.

### 3.6. GATA4

GATA4 functions as a crucial regulator of cardiomyocyte proliferation and differentiation. It exhibits high expression levels until birth and remains detectable in all cardiomyocytes [71]. It persists in its expression until approximately one week after birth, remaining easily detectable in cardiomyocytes and other cardiac cells, including those within the outflow tract (OFT), septa, and valves [71]. However, the absence of GATA4 in mice leads to embryonic lethality at E8.5, which is accompanied by cardia bifida, underscoring its crucial function in early heart formation [71,72]. Different studies have employed conditional knockout models. These models have shed light on GATA4’s dosage-sensitive role. Introducing LoxP sites to the GATA4 gene results in decreased expressions of around 20%, leading to structural heart defects [73]. Deleting GATA4 from cardiomyocytes using the Nkx2.5-Cre driver, occurring around E9.5, leads to myocardial thinning, the absence of mesenchymal cells in the endocardial cushions, a hypoplastic right ventricle, and embryonic lethality by E11.5 [74]. In contrast, the removal of Gata4 from endocardial cells through the Tie2-Cre driver results in embryonic lethality at E12.5 due to impaired epithelial-to-mesenchymal transformation (EMT), thus contributing to underdeveloped atrioventricular cushions [75]. Although GATA4 deletion when using the βMHC-Cre driver at E17.5 results in viable and fertile mice, it makes them susceptible to left ventricular dysfunction and dilation [76]. These findings underscore GATA4’s multifaceted role in regulating various aspects of cardiac development, including pacemaker cell development, as well as emphasize its importance in maintaining proper cardiac function.

On the other hand, GATA6 plays a pivotal role in the development of the SAN. Mutations in the GATA6 gene can lead to dysfunction in SAN patterning and size, ultimately contributing to the occurrence of arrhythmias [77]. This includes reduced levels of essential regulators for pacemaker cells like TBX3 and TBX5, which are accompanied by an increase in genes associated with the atria, such as Nkx2.5 and Nppa. Additionally, the arrangement of the SAN seems disturbed, particularly in the loss of HCN4+ pacemaker cells, which are mainly present in the head region [77]. While GATA6’s involvement in pacemaker cell differentiation is clear, it appears to have different functions depending on the type of cells within the SAN. In ISL1+ myocytes and HCN4+ conduction cells, GATA6 acts as an activator for the genetic program needed for pacemaker cell differentiation. It functions upstream of various transcriptional regulators in the SAN, including TBX3, TBX5, and TBX18 [77]. On the contrary, in endothelial cells, GATA6 probably regulates pacemaker cell differentiation indirectly, potentially through the influence on paracrine factors like EDN1, which plays a role in SAN cell differentiation [78]. An analysis of transcripts showed reduced levels of both EDN1 and one of its receptors, EDNRB, in the hearts of Gata6+/− mice as early as E11.5. This suggests that one way in which GATA6 contributes to the regulation of pacemaker cell differentiation in these cells is by modulating the activity of EDN1 [77].

### 3.7. HAND1

HAND1 plays a critical role in the specification and differentiation of embryonic structures, including the cardiac muscle of the heart [79]. It functions as an essential regulator for determining the fate of cardiac precursor cells, and it is involved in morphogenesis—a process controlled by the BMP signaling pathway [80]. Mutations in the HAND1 gene have been linked to congenital heart disease, highlighting its significance in heart development [81,82]. Moreover, recent research has revealed that BMP signaling can activate HAND1 regulation, further illuminating its role in heart muscle development [79].

### 3.8. IRX3

IRX3 plays a crucial role in regulating rapid electrical propagation within the ventricular conduction system by facilitating the transcription of Cx40 and Cx43 genes [83]. The development of the ventricular conduction system is tightly controlled by the activation of various transcription factors, including NKX2-5, TBX3, TBX5, and ID2 [42,83,84,85,86,87,88]. The dysregulation or loss of these transcription factors can result in a range of cardiac defects, particularly NKX2-5 and TBX5 loss, which can elevate the risk of arrhythmias [83]. IRX5 exhibits a gradient of expression within the ventricular myocardium, with the epicardium showing lower expression and the endocardium displaying higher expression levels [89]. Mutations in IRX5 are associated with an increased susceptibility to arrhythmias due to abnormal repolarization in the ventricular conduction system, which is influenced by the absence of a homeostatic Kv4.2 gradient [89,90].

The identification and understanding of the transcription factors involved in cardiac development, particularly in the context of the CCS, play a pivotal role in bridging the gap between existing knowledge and the practical applications in reprogramming strategies. Understanding the transcription factors’ roles in cardiac development provides a roadmap for designing targeted reprogramming approaches. By harnessing these insights, the efficiency and effectiveness of reprogramming strategies can be optimized, bringing us closer to the practical application of biological pacemakers and other therapeutic interventions for cardiac conduction disorders.

## 4. Key Signaling Pathways Involved in CCS Development

The conserved signaling pathways that have been found to be crucial for CCS specialization are Notch, BMP, Wnt, and NKX2-5 [37,87,91,92]. 

### 4.1. Notch Signaling

The Notch signaling pathway is crucial in determining cell fate and differentiation, along with shaping tissue patterns [91,93,94]. This evolutionarily conserved pathway is involved in various biological processes across different species. Knock-out studies have highlighted the significance of Notch1 in embryonic development. Notably, the knockout of Notch1 results in lethality around the E9.5 to E11.5 period due to its crucial role in the development of the sinus venous valve and the SAN [91,92]. These functions are executed by coordinating myocardial Wnt and NRG1 signaling processes [91,92]. Such insights underline the intricate orchestration of Notch signaling in cardiac development, as well as its critical role in ensuring proper heart formation and function.

### 4.2. BMP Signaling Pathway

The BMP (bone morphogenetic protein) signaling pathway is a critical orchestrator in the differentiation processes of both the SAN and the AVN, while also playing a pivotal role in regulating cardiac progenitor development [95]. The regulation of this pathway is intricately managed by the SMAD proteins, which act as essential mediators within the broader context of the BMP signaling cascade [96]. BMPs are categorized within the TGFβ (transforming growth factor-beta) superfamily [96]. The impact of the TGFβ pathway extends beyond the SAN and AVN differentiation, thereby encompassing a multitude of processes spanning the entirety of the heart muscle while also wielding significant influence over the intricate formation and precise patterning of the CCS [87,97]. TGFβ signaling plays a vital role in developing heart muscles and shaping the CCS, with *TGFβ1*, *TGFβ2*, and *TGFβ3* being expressed at specific stages and regions of CCS development [98].

### 4.3. Wnt Signaling

The Wnt signaling pathway regulates the proliferation and differentiation of cardiac progenitor cells during cardiac development and in the formation of the conduction system. Recently, Liang and colleagues have shown that canonical Wnt signaling promotes the pacemaker cell specification of the cardiac mesodermal cells derived from mouse and human embryonic stem cells [35]. They have shown that one of the key canonical Wnt/β-catenin ligand, Wnt3a, enhances the expression of a chamber of the cardiomyocyte gene *NKX2-5*. This raises the number of pacemaker-like myocytes while reducing cardiac troponin T-positive pan-cardiac differentiation [35]. The signaling pathways involved with the development of the CCS of the heart muscle interact with each other and with various other factors to regulate the development and function of the CCS. Reprogramming efforts in human-induced pluripotent stem cells have shown the impact of other signaling pathways like FGF and retinoic acid, which reprogram the cardiac mesoderm to generate SAN-like cells [99]. Transcriptome analyses of mouse and human sinoatrial node cells and sinoatrial ring (SAR) in zebrafish have revealed a conserved genetic program [35,39,40,99,100,101,102]. 

## 5. The Genetic Network of CCS Development

A unique gene expression mechanism enables cardiac pacemaker cells in the SAN to fire autonomously and initiate the heartbeat. The CCS is evolutionarily conserved in the building plan of the heart, and this indicates that the cellular and molecular mechanisms that drive the formation of pacemaker tissues are almost similar among vertebrates. Studies have shown that mammalian pacemaker CMs exhibit typical pacemaker action potentials and express molecular markers such as *Isl1, Shox2*, and *Hcn4* [67,101,103]. These mammalian genes are conserved in zebrafish and other teleost species. The knocking down of these genes in zebrafish leads to bradycardia, which is a phenotype indicating defects in cardiac pacemaker activity, thus reinforcing their vital roles in regulating pacemaker development [67,103,104]. The gene regulatory network (GRN) responsible for controlling CCS specification involves transcription factors (TFs) and signaling pathways. This network’s core components are the *cis-*acting regulatory regions that TFs bind to, which orchestrate the precise regulation of CCS development and function. Recent studies in mice have reported an *Isl1*-specific enhancer, which has not been identified in zebrafish [105]. However, there is limited understanding of the underlying gene regulatory network of these critical cells that are responsible for the heart’s electrical conduction. 

To gain a comprehensive understanding of these GRNs, it is essential to identify the *cis*-regulatory modules integral to the development of the CCS. These modules are pivotal in orchestrating the intricate molecular mechanisms responsible for regulating gene expression in the CCS. Specifically, tissue-specific gene expression patterns necessitate the presence of long-range regulatory regions, which are often referred to as enhancers. These enhancers are responsible for finely tuning the spatial, temporal, and dosage-dependent expression of target genes [106], thereby ensuring the precise and coordinated formation of pacemaker tissues within the heart. Elucidating the function and interactions of these *cis*-regulatory modules is crucial for unraveling the complexities of CCS development, as well as paving the way toward understanding the underlying molecular mechanisms that govern its expression and function.

The advancement of genomics technology and methodologies has provided valuable tools for dissecting the regulatory landscape of the CCS. The availability of publicly accessible genomics data and well-established techniques such as chromosomal conformation capture (3C, 4C-Seq, 5C, and Hi-Seq), along with newer approaches like FAIRE-Seq and ATAC-Seq, facilitates the identification of regulatory elements [42]. Additionally, single-cell sequencing and genome-wide ChIPseq datasets, combined with evolutionary conservation studies across vertebrate models (e.g., mice, chicken, and zebrafish), contribute to the discovery of multiple regulatory landscapes, including cardiac conduction-specific enhancers [42]. 

Single-cell RNA sequencing (scRNA-seq) and spatial transcriptomics (ST) offer valuable insights into the discovery of multiple regulatory landscapes for cardiac conduction-specific enhancers. ScRNA-seq helps understand the cellular changes that occur during heart development and diseases, while ST provides spatial context to gene expression profiles within the heart’s various structures [107]. These technologies bridge basic research and clinical applications, thereby aiding in the discovery of the key regulators of cardiac development and the mechanisms behind heart diseases. In clinical practice, scRNA-seq and ST can aid in tailoring treatments for heart diseases. By analyzing heart samples from different individuals, researchers can create a database of transcriptional changes before and after therapies [107]. This is especially relevant for chronic heart conditions and post-myocardial infarction prognosis. However, their application in arrhythmia diseases is currently limited.

Despite their promise, scRNA-seq and ST have challenges, including sample acquisition, data quality, and analysis complexity. Obtaining human heart tissue samples, both healthy and diseased, is challenging due to ethical considerations. Optimizing sample handling procedures, library preparation, sequencing depth, and data quality control processes are essential [107]. Additionally, refining bioinformatics analysis algorithms and reducing high sequencing costs are ongoing efforts in which to make these technologies more accessible to researchers.

The potent synergy of mathematical modeling and data integration emerges as a formidable force in predicting GRNs, thereby offering unparalleled insights into the intricate machinery that drives biological processes. These mathematical models span a spectrum of hypotheses, ranging from the driving M-clock models rooted in I_K2_ decay theories to the ascendancy of I_f_-based models triggered by the discovery of HCN channels [108]. The conceptual foundation of these models, such as the SD and ML models, is enriched by the assimilation of empirical data on sarcolemmal currents and Ca2+ cycling, which results in a nuanced interpretation of the intricate pacemaking machinery [108]. The remarkable adaptability of these models, informed by the incorporation of novel data, reflects a commitment to expanding our understanding of CCS function while also emphasizing the need for judicious model refinement over mere expansion [109]. 

**Figure 2:**
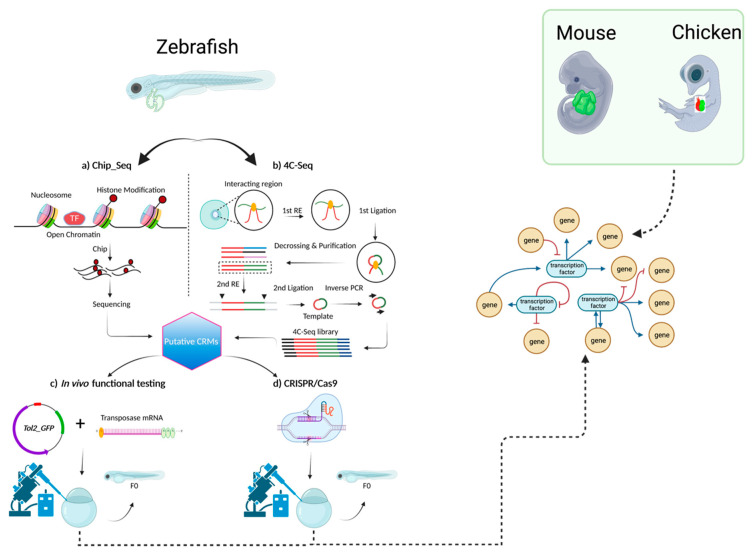
The proposed methodology for further investigating CCS-specific enhancers that will lead to building an informative GRN. Schematic of methods that can be used to identify *cis*-regulatory modules (CRMs) that use isolated hearts of various key developmental models (zebrafish, mice, or chicken). For each model, in this case zebrafish is shown, various chromatin capture methods like ChIP-Seq or 4C-Seq can be employed to obtain a list of putative CRMs. These CRMs can be functionally tested and investigated further using genome-editing techniques like CRISPR/Cas9. A functionally validated GRN can then be generated by intersecting data obtained from several developmental models.

However, the crux of this progress lies within this dynamic interplay of theoretical insights and experimental realities. The validation of enhancers through in vivo experimentation using cutting-edge techniques like CRISPR/Cas9-mediated mutant generation is pivotal (Figure 2). This process not only unravels the roles enhancers play in CCS development, but also illuminates how deviations from normal gene expression patterns can lead to disruptive conditions such as cardiac arrhythmias. Significantly, the construction of a comprehensive GRN that encompasses key transcription factors (TFs) like NKX2-5, TBX3, TBX5, ISL1, GATA4, GATA6, HAND1, SHOX2, IRX3, and IRX5 stands as an imperative undertaking. Systematically deciphering the intricate interactions between these TFs and the identified enhancers promises to unveil the hidden dynamics shaping CCS development. As the veil lifts, potential therapeutic avenues for cardiac disorders come into view, and these are propelled by an amalgamation of computational insights and empirical validation. Through the convergence of mathematical modeling, data integration, and functional experimentation, the journey toward unraveling the regulatory complexities governing cardiac function gains momentum, thereby ultimately yielding a profound comprehension of this quintessential biological process.

## 6. Conclusions and Future Directions

The intricate landscape of cardiac pacemaker cell development is central to the quest for biological pacemakers. Reprogramming existing cardiac cells into pacemaker-like entities represents a multifaceted endeavor, one that requires precise control over gene expression and signaling pathways. 

Understanding the molecular intricacies governing pacemaker cell development is paramount as it unveils potential targets for genetic manipulation. While significant progress has been made in understanding the signaling pathways and transcription factors involved in pacemaker cell development, there is a pressing need to delve deeper into the upstream regulatory networks. The path to harnessing the potential of biological pacemakers hinges on our ability to predict, manipulate, and control these regulatory networks. 

Looking ahead, the future of cardiac research should emphasize several key directions. First, there is a crucial need to integrate cutting-edge computational tools, publicly available databases, and mathematical modeling to predict and understand these complex regulatory networks. These tools will serve as the foundation for the development of CCS-specific gene regulatory networks (GRNs). 

Second, the in vivo functional testing of regulatory elements, including knock-out studies, will be pivotal in confirming the predictions generated through computational models. This experimental validation will bridge the gap between theoretical insights and practical applications.

Finally, fostering extensive interdisciplinary collaboration among experts in system biology, developmental biology, molecular biology, and computational science is essential. Together, these diverse perspectives and skill sets will facilitate a comprehensive understanding of CCS development and the realization of biological pacemakers.

## Data Availability

No new data was created.
